# Supplementary material for: Natural Fiber-Reinforced Foamed Rubber Composites: A Sustainable Approach to Achieving Lightweight and Structural Stability in Sole Materials
Source: Polymers (Basel). 2025 Jul 26;17(15):2043. doi: 10.3390/polym17152043 (PMC12349017; doi:10.3390/polym17152043)
Supplement: Supplementary file 1 [file polymers-17-02043-s001.zip › polymers-3749836-supplementary.pdf]

polymers

MDPI

Supplementary Information

Natural Fiber-Reinforced Foamed Rubber Composites: A Sustainable Approach to Achieving Lightweight and Structural Stability in Sole Materials

Yi Jin [a], Shen Chen [a], Jinlan Xie [b], Weixing Xu [b,d]*, Yunhang Zeng [b,c] and Bi Shi [b,d]

[a] College of Biomass Science and Engineering, Sichuan University, Chengdu 610065, China.
[b] National Engineering Laboratory for Clean Technology of Leather Manufacture, Sichuan University, Chengdu 610065, China.
[c] Key Laboratory of Leather Chemistry and Engineering, Sichuan University, Ministry of Education, Chengdu 610065, China.
[d] Research Center for Biomass Materials, Tianfu Yongxing Laboratory, Chengdu 610213, China
* Correspondence: e-mail: xuwx@scu.edu.cn

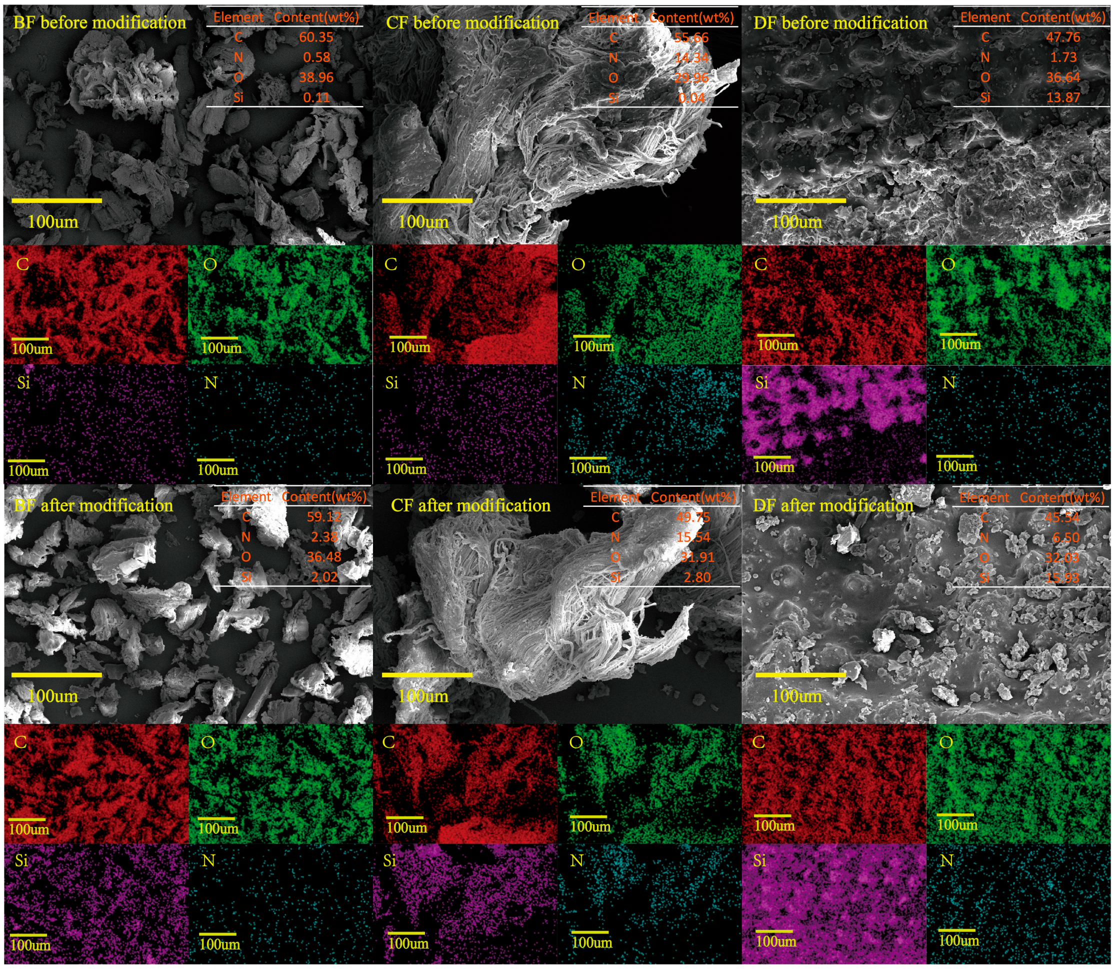

Figure S1. SEM and EDS elemental images of natural fibers.

Figure S1 presented the SEM and EDS elemental mapping images of three types of fibers, both before and after modification with KH550. Before modification, BF contained negligible amounts of N and Si elements. In contrast, following modification, the content of N and Si elements in BF increases significantly. For CF, which was inherently a protein-

based material, its N content before modification was relatively high, but no Si content. After modification, the Si content in CF increased significantly. As for DF, it has a high silicon content but no nitrogen content. After modification, the nitrogen content in DF increased markedly. These results collectively demonstrated the successful grafting of KH550 onto natural fibers. Additionally, the EDS spectra revealed that the N and Si elements were essentially uniformly distributed on the modified fibers, indicating that KH550 was evenly coated on the surface of natural fibers.

Table S1. Mechanical properties of NR and its composites at different fiber contents.

Sample	Fiber contents (wt%)	Tensile strength (MPa)	Elongation at break (%)	Tear Strength (N/mm)
Blank	0	8.47 ± 0.05	687.3 ± 226.7	50.36 ± 11.06
AC-NR	0	5.24 ±1.36	369.8 ± 67.4	32.95 ± 5.82
BF-NR	3	6.56 ± 0.19	400.5 ± 33.0	34.87 ± 3.92
	5	6.89 ± 0.35	430.3 ± 22.4	40.45 ± 4.34
	10	7.47 ± 0.47	560.2 ± 35.6	43.60 ± 3.23
	15	8.22 ± 0.21	680.0 ± 30.6	39.95 ± 4.88
	20	8.23 ± 0.56	638.0 ± 20.0	44.60 ± 5.73
	25	6.45 ± 0.08	580.4 ± 11.1	36.10 ± 3.93
	30	5.89 ± 0.31	420.2 ± 20.0	37.60 ± 5.85
	40	4.30 ± 0.98	300.5 ± 25.0	29.87 ± 3.99
CF-NR	3	6.73 ±0.22	455.0 ± 64.0	34.04 ± 4.16
	5	7.35 ±1.60	516.4 ± 40.0	33.58 ± 4.52
	10	9.99 ±0.60	653.8 ±55.3	37.70 ± 3.45
	15	10.25 ±0.28	753.2 ± 48.9	47.03 ± 5.07
	20	10.13 ±1.38	745.5 ± 69.1	48.01 ± 1.88
	25	8.15 ±0.05	694.4 ± 35.3	51.15 ± 4.20
	30	8.97 ±0.28	462.0 ± 46.0	47.65 ± 2.05
	40	8.72 ±1.32	366.5 ± 36.5	39.98 ± 3.45
DF-NR	3	6.42± 0.17	390.0 ± 30.5	33.50 ± 3.79
	5	6.74 ± 0.33	415.8 ± 20.0	39.00 ± 4.12
	10	8.30 ± 0.45	545.7 ± 32.0	42.30 ± 3.03
	15	8.52 ± 0.20	665.5 ± 28.0	48.60 ± 4.62
	20	8.66 ± 0.54	665.7 ± 18.5	43.70 ± 5.4
	25	6.38 ± 0.07	565.9 ± 10.0	34.80 ± 3.70
	30	5.70 ± 0.29	405.7 ± 18.5	46.30 ± 5.69
	40	5.41 ± 0.17	320.5 ± 22.1	28.50 ± 3.87

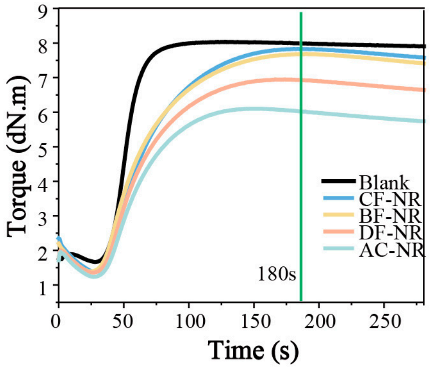

Figure S2. The vulcanization curves of pure NR and composites.

Section S1 Characterization parameters for the structure and properties of materials

1.1. The analysis of the creep-recovery behavior using the Burger's model and the Weibull distribution.

The creep deformation of materials can be divided into three categories (Fig. S1): elastic deformation, $\mathcal{E}_{SM}^{*}$ $\mathcal{E}_{SM}^{*}$ $\mathcal{E}_{SM}^{*}$represents$\mathcal{E}_{KV}^{*}$ elastic $\mathcal{E}_{KV}^{*}$deformation, $\mathcal{E}_{KV}^{*}$ represents viscous deformation, and $\mathcal{E}_{\infty}^{*}$ represents irreversible deformation. In addition, $\mathcal{E}_{MAX}^{*}$ represents the maximum deformation. $\mathcal{E}_{R}^{*}$ represents recoverable deformation. θ_{SK}^{*} represents the proportion of elastic deformation and viscous deformation in the total deformation [51,52]. The relationship of the parameters is expressed as Equation (1) (2) (3):

$$\mathcal{E}_{R}^{*} = \mathcal{E}_{SM}^{*} + \mathcal{E}_{KV}^{*} \quad (1)$$

$$\mathcal{E}_{MAX}^{*} = \mathcal{E}_{SM}^{*} + \mathcal{E}_{KV}^{*} + \mathcal{E}_{\infty}^{*} \quad (2)$$

$$\theta_{SK}^{*} = \mathcal{E}_{R}^{*}/\mathcal{E}_{MAX}^{*} \times 100\% \quad (3)$$

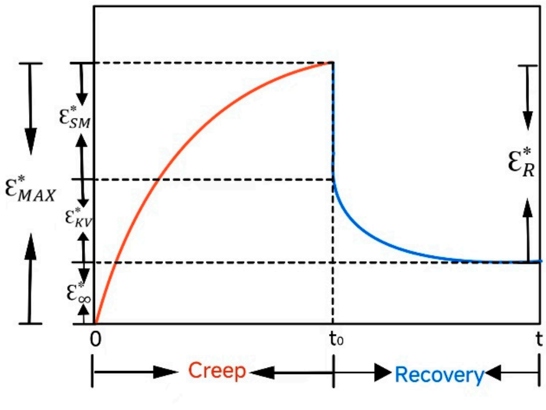

Figure S3. Schematic diagram of creep-recovery curve.

The Burger's model and the Weibull distribution equation are classic models for studying the creep-recovery behavior of materials. These two models can be used to further analyze the creep-recovery behavior of materials.

(1) Burger's Model

The Burger's model is composed in series of the Maxwell and Kelvin–Voigt models. It is often used to simulate the viscoelastic behavior of materials[53-55]. The creep deformation ε(t) of viscoelastic materials is expressed as Equation (4).

$$\mathcal{E}(\text{t}) = \sigma_0/E_M + \sigma_0/E_K\,(1 - \exp\,(-\,\text{t}\,E_K/\eta_K)) + \sigma_0/\eta_M\,\text{t} \quad (4)$$

In the equation, σ_0 represents the constant stress applied in the creep test, E_M and E_K represent the elastic moduli of the Maxwell and Kelvin–Voigt elements respectively, η_M and η_K represent the viscosities of the Maxwell and Kelvin-Voigt dampers respectively. The ratio of η_K to E_K is called the relaxation time τ (The value is given by Equation (5).

$$\tau = \eta_K/E_K \quad (5)$$

(2) Weibull Distribution Equation

The Weibull distribution equation is widely used to describe the recovery behavior of viscoelastic materials, as shown in Equation (6) (7) [55,56]:

$$\mathcal{E}_{RE}(t) = \mathcal{E}_{KV}\left[\exp\left(-((\mathrm{t}-\mathrm{t}_0)/\eta_r)^{\beta_r}\right)\right] + \mathcal{E}_{\infty} \tag{6}$$

$$\mathcal{E}_{SM} = \mathcal{E}_{MAX} - \mathcal{E}_{KV} - \mathcal{E}_{\infty} \tag{7}$$

In the equation, $\mathcal{E}_{RE}(t)$ represents the recovery deformation that changes with time when the stress is released, $\mathcal{E}_{KV}$ represents the viscoelastic deformation, which is determined by the characteristic time (η_r) and shape factor (β_r) within the recovery time, t represents the recovery time, and t_0 ($t_0 = 1800$ s) represents the time when the stress is released.

The physical meanings of the parameters in Burger's model and Weibull fitting are as follows:

η_M represents the control over the steady-state creep rate and irreversible viscous flow of the material. It reflects the ease with which molecular chains or defects within the material undergo relative slippage and rearrangement. The larger η_M is, the smaller the steady-state creep rate becomes, and the stronger the material's resistance to permanent deformation.

E_K represents the delayed elastic response or recoverable creep component of the material. It reflects the ability of secondary structures within the material (such as molecular chain entanglements or microcrystalline regions) to actively resist deformation while facilitating recovery. The larger E_K is, the smaller the final magnitude of delayed elastic deformation becomes.

τ characterizes the time required for delayed elastic deformation to reach its equilibrium value, serving as a key parameter quantifying the speed of the delayed response. It directly reflects the characteristic temporal scale of rearrangement or motion within secondary structures.

E_M represents the instantaneous elastic response of the material. When stress σ_0 is abruptly applied, strain immediately exhibits a step change of σ_0/E_M. This reflects the capability of molecular bonds or crystal structures within the material to resist transient deformation. The larger E_M becomes, the smaller the instantaneous deformation.

η_K It controls the rate of development of delayed elastic deformation. It reflects the rate at which secondary structures within the material rearrange or overcome energy barriers to achieve elastic deformation. The larger the η_K (or the larger the τ), the slower the development of delayed elastic deformation.

The shape parameter β_r controls the morphology of the distribution curve, reflecting the uniformity or dispersion of recovery times: Higher β_r values indicate a more uniform recovery process. Lower β_r values signify greater dispersion in recovery times. The scale parameter η_r represents the characteristic time of the recovery process, corresponding to the typical time required for the material to complete recovery. The location parameter E_{KV} shifts the distribution curve along the time axis, defining the minimum recovery time or initial delay before the material's recovery initiates.

1.2. Detailed parameters for XPS testing:

Excitation source: Al Kα ($h\nu = 1486.8$ eV)
Beam spot: 400 μm
Analysis chamber vacuum: 2×10^{-9} mbar
Operating voltage: 15 kV
Filament current: 10 mA
Survey scan: pass energy 150 eV, step size 1 eV
High-resolution scan: pass energy 50 eV, step size 0.1 eV

Each high-resolution spectrum was accumulated over at least five sweeps (the exact number varied by element).

Energy-scale calibration: referenced to C 1s at 284.8 eV.

1.3. Methods for crosslinking density testing

The crosslink density of NR and its composites was determined using the equilibrium solvent swelling method. First, cubic samples with dimensions of 10 mm × 10 mm × 1 mm were completely immersed in toluene and kept in a water bath at 25°C for 48 h. After that, the samples were taken out of the toluene, and the residual toluene on the surface was immediately wiped off. The samples were subsequently weighed with precision to determine their mass m_g. Subsequently, the samples were dried in a 60°C forced-draft oven for 36 h and weighed accurately again to obtain the mass m_s.

$$V_{2m}(t) = 1/(1 + G) \quad (8)$$

$$G = (m_g - m_s)/m_s + \rho_e/\rho_s \quad (9)$$

In this context, ϱ_e and ϱ_s denote the densities of the elastomer and the solvent (0.865 g/cm^3 for toluene), respectively. The density of the elastomer samples was determined by hydrostatic weighing in accordance with ISO 2781:2018.

The crosslink density of the samples (v) was calculated using the Flory-Rehner equation [57], based on measurements in toluene solvent:

$$v = -[\ln(1 - V_{2m}) + V_{2m} + X_{12}V_{2m}^2]/[V_1 V_{2m}^{\frac{1}{3}} - V_{2m}/2] \quad (10)$$

Here, V_1 is the molar volume of the solvent (106.5 cm^3/mol for toluene), V_{2m} is the volume fraction of polymer in the sample at equilibrium swelling, and X_{12} is the Flory-Huggins polymer-solvent interaction parameter (with a value of 0.393 for toluene).

Table S2. Density of pure NR and composites.

	Mass/ g	Length/ mm	Width/ mm	Height/ mm	Volume/ mm^3	Density/ g/cm^3	
	2.807	16.97	15.46	11.19	2935.766	0.956	
Blank	2.777	19.07	15.28	10.01	2916.810	0.952	0.952
	2.712	18.30	15.26	10.23	2856.809	0.949	
	2.400	17.03	11.73	20.07	4009.221	0.599	
AC-NR	2.020	17.19	12.47	17.68	3789.872	0.533	0.535
	3.170	17.68	18.05	20.97	6692.030	0.474	
	2.691	17.14	15.16	15.86	4121.100	0.653	
BF-NR	2.802	15.85	14.91	16.50	3899.338	0.719	0.712
	2.663	15.71	15.34	14.43	3477.506	0.766	
	2.744	16.92	12.84	16.96	3684.607	0.745	
CF-NR	3.715	18.54	16.51	17.85	5463.803	0.680	0.721
	2.896	17.40	14.88	15.17	3927.695	0.737	
	1.830	16.59	10.90	16.39	2963.820	0.617	
DF-NR	1.315	13.63	11.16	14.69	2234.508	0.588	0.633
	1.785	14.25	12.78	14.13	2573.285	0.694	

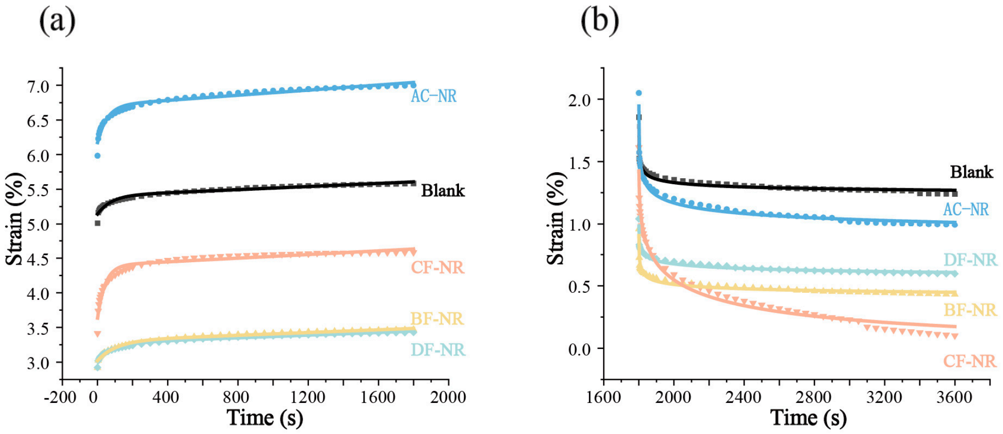

Figure S4. a. Creep fitting curves of pure NR and composites using Burger's model; b. Recovery fitting curves of pure NR and composites using the Weibull distribution function.

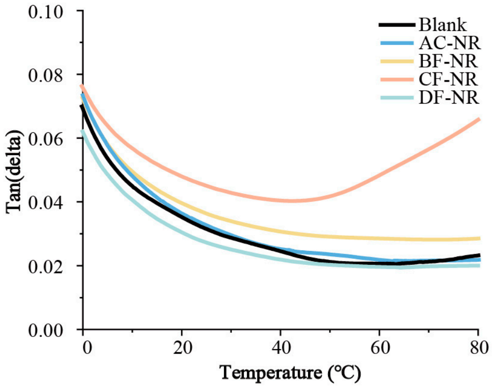

Figure S5. Loss factor curves of pure NR and composites.

The loss factor is the ratio of the loss modulus to the energy storage modulus, which indicates the ability of a material to dissipate energy and is dimensionless in magnitude

Table S3. The relationship between the peak positions in the FT-IR spectrum and the functional groups.

Wavenumber (cm^{-1})	Assignment [41][42]
3300	O-H stretching
2960	C-H stretching (CH_3)
2916	C-H stretching (CH_2)
2850	C-H sym. stretching
1662.73	C=O stretching
1583.7	C=C stretching

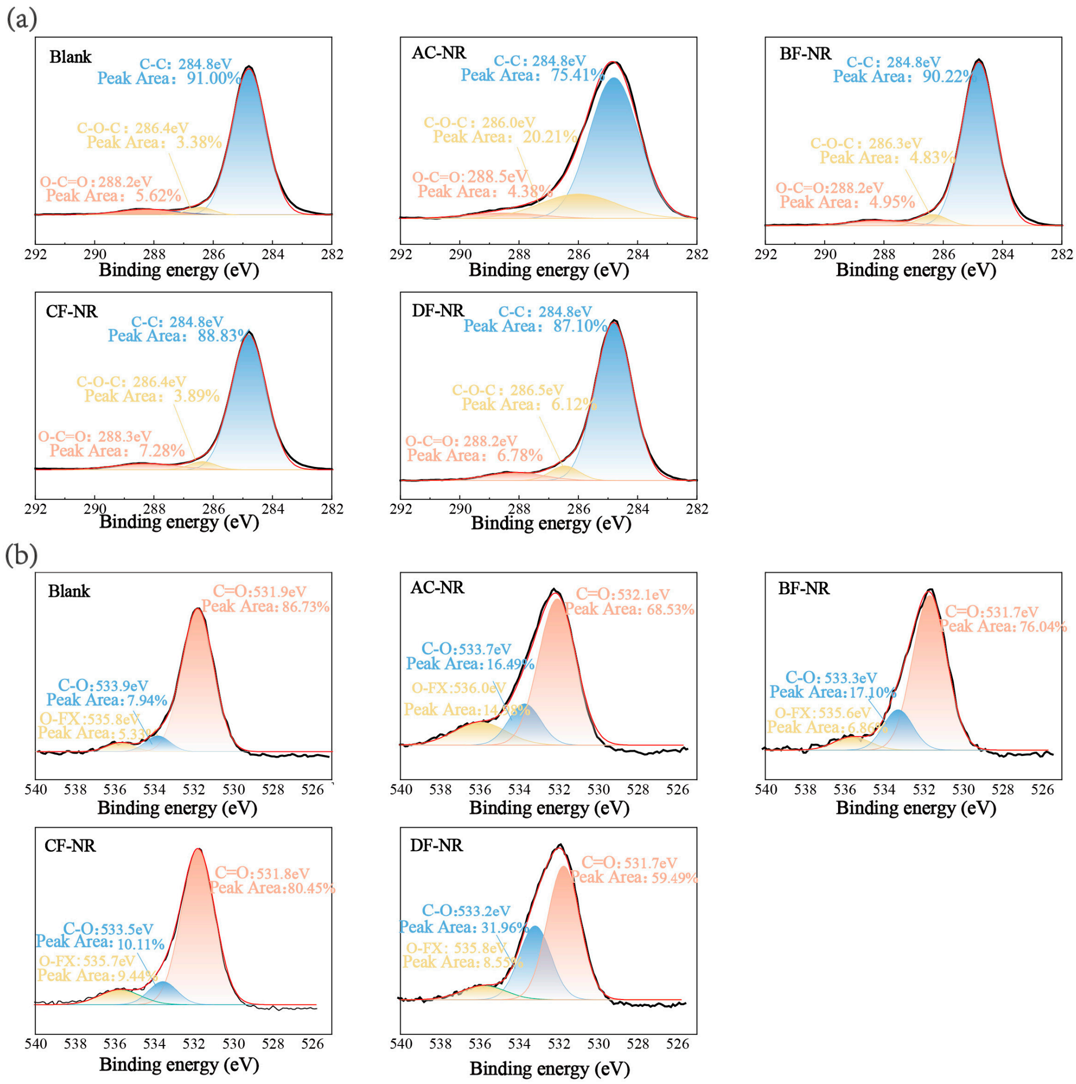

Figure S6. X-ray Photoelectron Spectroscopy (XPS) spectra of pure NR and composites (a. XPS C1s; b. XPS O1s).

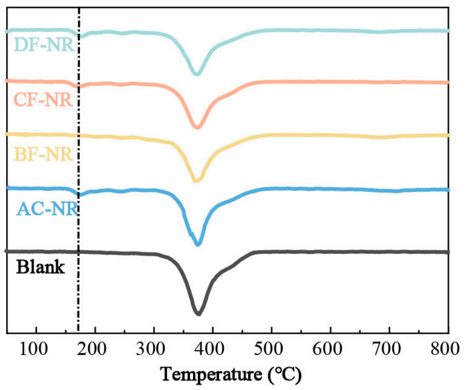

Figure S7. Derivative Thermogravimetric (DTG) curves of pure NR and composites.

Table S4. Parameters corresponding to the TG curves of pure NR and composites.

Sample	Tonset[1]	Tmax1[2]	Tmax2[2]	Tmax3[2]	Char yield at 800 °C/%
Blank	169.6	188.9	374.4	-[3]	0.77
AC-NR	152.3	177.7	370.3	705.4	7.88
BF-NR	168.6	179.1	372.4	677.3	5.37
CF-NR	167.4	167.4	367.8	688.8	7.42
DF-NR	167.4	178.1	369.3	676.3	6.37

[1] Tonset is the temperature calculated from the tangent line at the inflection point of the TGA curve. [2] Tmax refers to the temperature at which the rate of mass loss during material decomposition reaches its maximum value. [3] "-"indicates that it was not observed in the image.

The absorption peak at 1500-1750 cm^{-1} in the infrared region changed after the addition of AC. After the addition of AC, the absorption peak at 1538 cm^{-1} in the material disappeared, and a new absorption peak at 1596 cm^{-1} emerged. This is due to the incomplete decomposition of AC. Fig. S6 shows the incomplete decomposition process of AC.

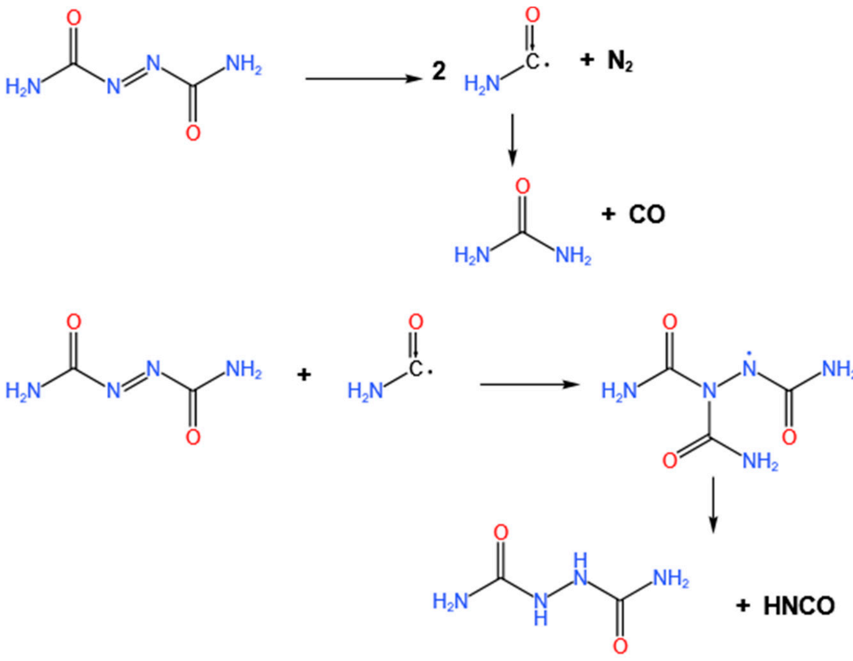

Figure S8. The incomplete decomposition process of AC.

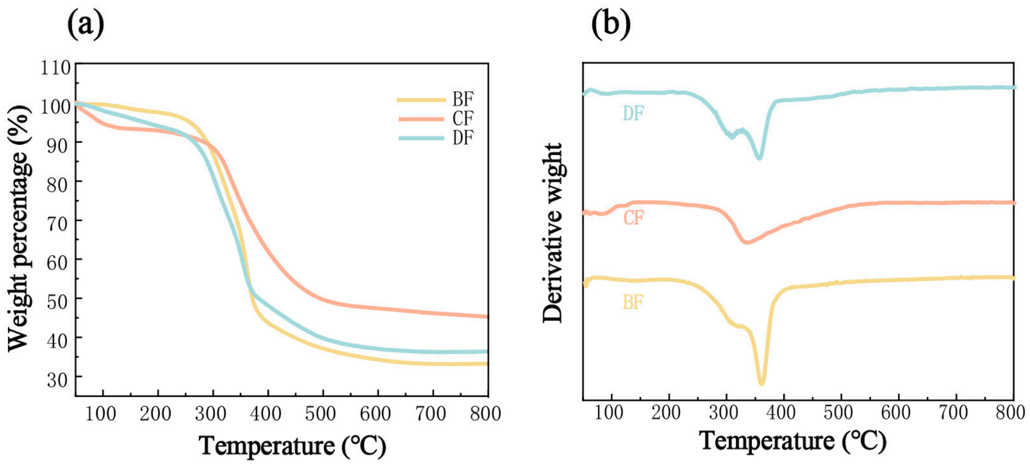

Figure S9. Thermogravimetric analysis of the modified fibers. a, TGA; b, DTG.

The pure fibers were tested as control groups to evaluate their individual thermal stability. The results indicated that none of the natural fibers degraded during composite processing. And the results also confirmed that natural fibers possessed the characteristic of high char yield

Table S5. Mechanical properties of pure NR and composites before and after aging.

Sample	Blank	AC-NR	BF-NR	CF-NR	DF-NR
Tensile strength before aging / MPa	8.47±0.05	5.24±1.36	8.23±0.56	10.13±1.38	8.66±0.54
Tensile strength after aging /MPa	0.58±0.09	0.75±0.09	1.44±0.30	1.92±0.09	0.96±0.02
Tensile strength retention rate / %	6.79	14.39	17.5	18.99	11.14
Tear strength before aging/ N/mm	50.36±11.06	32.95±5.82	44.60±5.73	48.01±1.88	43.70 ± 5.4
Tear strength after aging /N/mm	1.10±0.05	2.89±0.45	5.74±0.24	4.00±0.39	5.96±0.59
Tear strength retention rate / %	2.18	8.78	13.04	8.33	13.64
Elongation at break before aging (%)	687.3±226.7	369.8 ± 67.4	638.0 ± 20.0	745.5 ± 69.1	665.7 ± 18.5
Elongation at break after aging (%)	152.0±22.1	155.4±42.0	59.1±9.3	53.6±7.2	87.4±13.1

Table S6. Crosslinking degree and crosslinking densities before and after aging.

Sample	Before Aging		After Aging	
	Crosslinking degree	Crosslinking densities (10^{-4} mol/cm^3)	Crosslinking degree	Crosslinking densities (10^{-4} mol/cm^3)
Blank	100.00	1.295	100.00	0.719
AC-NR	96.93	1.030	91.25	0.708
BF-NR	100.00	2.774	97.21	1.937
CF-NR	100.00	3.671	100.00	2.120
DF-NR	100.00	2.061	96.98	1.649

References

51. Jing, Q.; Liu, Q.; Li, N.; Dong, Z.; Silberschmidt, V.V. Effect of graphene-oxide enhancement on large-deflection bending per-formance of thermoplastic polyurethane elastomer. Compos. Part B 2016, 89, 1–8. http://doi.org/10.1016/j.compositesb.2015.11.033

52. Whba, R.; Su'ait, M.S.; Whba, F.; Sahinbay, S.; Altin, S.; Ahmad, A. Intrinsic challenges and strategic approaches for enhancing the potential of natural rubber and its derivatives: A review. Int. J. Biol., Macromol. 2024, 276, 133796. http://doi.org/10.1016/j.ijbiomac.2024.133796

53. Luo, R.K.; Zhou, X.; Tang, J. Numerical prediction and experiment on rubber creep and stress relaxation using time-dependent hyperelastic approach. Polym. Test. 2016, 52, 246–253. http://doi.org/10.1016/j.polymertesting.2016.03.026

54. Sandström, R. Basic analytical modeling of creep strain curves. Materials 2023, 16, 3542. http://doi.org/10.3390/ma16093542

55. Kamarian, S.; Song, J.I. Review of literature on eco-friendly sandwich structures made of non-wood cellulose fibers. J. Sandw. Struct. Mater. 2022, 24, 1653–1705. http://doi.org/10.1177/10996362211062372

56. Luo, S.; Tian, J.; Liu, Z.; Lu, Q.; Zhong, K.; Yang, X. Rapid determination of styrene-butadiene-styrene (SBS) content in modified asphalt based on Fourier transform infrared (FTIR) spectrometer and linear regression analysis. Measurement 2020, 151, 107204. http://doi.org/10.1016/j.measurement.2019.107204

57. Deka, R.; Sarmah, J.K.; Baruah, S.; Dutta, R.R. An okra polysaccharide (Abelmoschus esculentus) reinforced green hydrogel based on guar gum and poly-vinyl alcohol double network for controlled release of nanocurcumin. Int. J. Biol. Macromol., 2023, 234, 123618. https://doi.org/10.1016/j.ijbiomac.2023.123618.
